# Gender in public health research: Reflections on design and process across four research projects in low-and middle-income countries

**DOI:** 10.1371/journal.pgph.0000808

**Published:** 2023-04-12

**Authors:** Marta S. Palmeirim, Séverine Erismann, Andrea Leuenberger, Monica Berger-González, Sally Mtenga, Somphou Sayasone, Peter Odermatt, Helen Prytherch, Claire Somerville

**Affiliations:** 1 Swiss Tropical and Public Health Institute, Basel, Switzerland; 2 University of Basel, Basel, Switzerland; 3 University of the Valley of Guatemala, Guatemala City, Guatemala; 4 Ifakara Health Institute, Dar Es Salaam, Tanzania; 5 Lao Tropical and Public Health Institute, Ministry of Health, Vientiane, Lao People’s Democratic Republic; 6 Graduate Institute of International and Development Studies, Geneva, Switzerland; University of British Columbia, UNITED KINGDOM

## Abstract

A growing body of work clearly documents the gendered inequalities in health. The COVID-19 pandemic further exposed these deep inequities: men appear to be more vulnerable to poorer outcomes, but most of the global health workforce is female who are at increased risk of exposure to hospital infection. However, researchers often fail to adequately embed gender as part of the public health research. This paper reports findings from a synthesis exercise that identified some of the challenges of integrating gender in the design and processes of research studies in four projects conducted in six low- and middle-income countries. Through a collective retrospective meta-synthesis process with researchers from each project, we identified two main themes; (i) we deep dive on two of the structural pillars of conducting public health research (design and process) and (ii) we describe some of the underlying opportunities and resistances to the integration of a gender perspective in these research projects. In conclusion, we suggest that public health funding bodies require researchers to integrate gender in public health research from early on as part of the design and to conduct gendered analysis, as part of the overall drive towards more equitable health systems delivery.

## Introduction

It is well known that health systems are gendered with consequences that impact health system needs, experiences and outcomes at all levels [1–3]. However, gender is often neglected in public health research [4]. The COVID-19 pandemic, for example, exposed the deep fissures of gender inequality and inequity of health across the globe [5], making this a critical moment to reflect on how we address gender in all our public health research activities and practices. The pandemic struck in the year of the 25^th^ anniversary of the United Nations (UN) Beijing Declaration and Platform for Action that promised prioritization of gender mainstreaming in the policies, practices and programmes of the UN and its agencies [6]. Beijing set forth a number of strategies that sought to ensure the cross-sectoral integration of gender terminology and responsiveness somewhat assuming the world knew what that might mean in practice [7]. Even though the Beijing Declaration has been successful in including sex and gender analysis in research, producing knowledge on certain factors affecting women’s health [8] and raising awareness of the health effects of sex and gender, these still fail to address larger structural causes of health inequality among women. In the research realm, although research grant applications and, very occasionally, research ethics boards, request that applicants prepare responses to anticipated gender issues in their research, there are currently no mandatory obligations to collect or analyse gender data. Suffice to say, typically, study designs in public health research do not plan for the collection and analysis of gender data, especially in quantitative studies that use secondary data where researchers are at least one-step removed from data capture and rely on gender aggregated data sets.

As public health researchers, it is imperative that we reflect and act upon these shortcomings so public health research not only continues with Beijing-triggered mainstreaming, but also explores other determinants of health inequality within the intersectional social context of women’s lives, socioeconomic class, race and ethnicity and education level, among many factors [9]. In response to these shortcomings, international commitments to gender and health followed in the UN Millennium Development Goals (MDGs) and the 2015 Sustainable Development Goals (SDGs), progressively advanced global commitments to gender equality, health and well-being for all and underpin universal health coverage. The early uptake of these global level political commitments in the public health research sphere varied and typically addressed issues of systemic discrimination and representation and the neglected area of women’s health [10, 11].

Whilst such high-level activity are important policy levers in public health, at its core, much of public health, and certainly our research, is located at the grassroots where communities interface with health systems and practitioners. It is often at ground level where researchers in public health conduct their data collection with the aim to produce knowledge and evidence that advance health outcomes. These are spaces where the hand of gender mainstreaming rarely touches and where all too often gendered bias, roles and norms produce unintended consequences that may even be harmful [12–14].

In this paper, as public health researchers conducting empirical studies engaged with health systems, workforces, communities and patients, working on different longitudinal global public health research projects conducted between 2015 and 2021, we have engaged in a meta-synthesis process to reflect on how our respective projects engaged with gender throughout the research process. All four projects are funded under the same stream, the Swiss Programme for Research (r4d) on Global Issues for Development initiated by the Swiss National Science Foundation [15]. The r4d Programme aims to create and disseminate scientific knowledge and research-based solutions to local and national health issues in the global health context of the 2030 Agenda for Sustainable Development. This funding stream has proactively encouraged cross collaboration between research projects as part of a synthesis initiative to promote and disseminate research findings and learnings beyond individual r4d projects into a larger body of knowledge in the science-society-policy interface.

The first case study addresses health systems governance for equitable social health protection in Tanzania. The second investigates the health impacts of large natural resource extraction projects in Ghana and Tanzania on local communities. The third case study focuses on early detection of liver flukes in Lao People’s Democratic Republic (Lao PDR). The fourth and last case study explores surveillance and response to zoonotic diseases in Maya communities of Guatemala.

This paper is the result of the success of a synthesis dialogue drawing on experience from these four projects with whom the authors are researchers or part of the synthesis mandate. We report key findings in understanding how the different research projects addressed issues around gender including challenges and opportunities that gender perspectives play in public health research more widely. We describe two main themes, the first being two of the structural pillars of conducting public health research—design and process [16] and the second being some of the underlying opportunities and resistances to the integration of a gender perspective in these projects–with case study material illustrating experiences and learnings on how to move forward with a gender lens in public health research.

## Materials and methods

### Ethics statement

All interviewees provided verbal consent as to their participation in this synthesis mandate to share knowledge through a webinar and interviews with the intent to produce a policy brief and a scientific publication–the current manuscript. All interviewees were informed that they could withdraw at any moment without consequences.

All projects included in this meta-synthesis obtained the necessary ethical approvals. For the project on health systems governance for equitable social health protection in Tanzania, ethical approval in Tanzania was obtained from the institutional review board (IRB) of the Ifakara Health Institute (reference number: IHI/IRB/No: 35–2020) and the National Institute for Medical Research of Tanzania (NIMR; reference number: NIMR/HQ/R.8a/Vol. IX/3518). For the project on health impacts of large natural resource extraction projects in Ghana and Tanzania on local communities, ethical approval in Ghana was obtained from the Ghana Health Service Ethics Review Committee (reference number: GHS-ERC016/02/2019), in Tanzania from the IRB of the Ifakara Health Institute (reference number: IHI/IRB/No: 32–2018) and NIMR (reference number: 2969), and in Switzerland from the Ethics committee of Northwestern and Central Switzerland (EKNZ; reference number: 2018–00386) and the IRB of the Swiss Tropical and Public Health Institute (Swiss TPH). For the project on early detection of liver flukes, ethical approval was obtained in Lao PDR from the National Ethics Committee for Health Research (reference number: 98/NECHR) and in Switzerland from the EKNZ (reference number: R-2017-00869). Finally, for the project on surveillance and response to zoonotic diseases in Maya communities of Guatemala, ethical approval was obtained in Guatemala from the Ethics Committee Review Board from the Center for Health Studies, Universidad del Valle de Guatemala (reference number: 154–09–2016) and in Switzerland from EKNZ (reference number: 2016–00422).

### Study design

The study draws on four different six-year long studies conducted in six low-and middle-income countries with principal and co-investigators and their teams based in each country and in Switzerland. All the projects are interdisciplinary, multicentre north-south and south-south partnerships guided by an overarching vision of conducting research for development with pathways to impact. None of these r4d projects was designed as a “gender” project *per se* but all had completed the necessary funding application question that asked if the project would address gender issues and all gave affirmative responses.

For the current manuscript, we designed and implemented a collective retrospective meta-synthesis process engaging project researchers (and authors in this paper) to reflect on a set of key synthesis research questions (S1 Table). The methodology adapts a similar successful approach to r4d synthesis research [17]. We conducted semi-structured peer-interviews with principal investigators and their research partners. Each of the four research projects constitutes a case study. Researchers from each case study responded to three reflexive research questions (see Supporting Information) drawing on project documents, discussions and recollections of the research process. Each case study team produced a detailed PowerPoint presentation with collated responses. A public webinar held in November 2019 provided the opportunity to share and discuss the findings before drafting a synthesis White Paper (S2 Table). Detailed notes were taken during interviews and the webinar and all authors contributed to shared written materials collated and revised throughout the process. Our analysis is inspired by a reflexive methodology [18] whereby the researcher as interpreter is actively present, therefore affording us a group of researchers to engage in this retrospective synthesis.

## Results

During our analysis, we identified two main themes from our retrospective reflection across the four public health projects conducted in six LMICs: Burkina Faso, Ghana, Guatemala, Lao PDR, Mozambique and United Republic of Tanzania. The first theme centres on aspects of the conduct of research, namely research design and research process. The second theme we present revolves around resistances and opportunities, such as *attitudes* towards the integration of gender dimensions to public health research, sometimes in the face of resistance from colleagues and wider stakeholders who do not see the relevance of gender to the project aims. Our interpretation of each of these the two themes are presented below, along with how the four example r4d projects encountered these themes. Note that we adopted the WHO’s definition of sex and gender; “gender refers to the characteristics of women, men, girls and boys that are socially constructed”, whereas sex “refers to the different biological and physiological characteristics of females, males and intersex persons, such as chromosomes, hormones and reproductive organs” [19].

### Research design and process

It is critical to build gender within the conceptualization and design of the research study, its methodology and methods at the outset [20]. Even when projects do not hypothesize gender dimensions to their analysis and findings, gensuring that gender data is collected at least enables researchers to extract and later conduct a gender analysis offering the opportunity for post-study exploration of findings. This first case study serves as an example of a study that integrated the collection of sex-disaggregated data [21] from early on at the design phase.

### Opisthorchiasis in Lao PDR: A case study

The study aimed to assess liver morbidity currently existing in the rural Lao communities and understanding the contribution of the liver fluke infection for it. It followed-up patients with liver morbidity to understand the outcome after treatment, and provided access to adequate management and clinical support in health care. Moreover, the research accompanied the establishment of a biobank and work on biomarkers for early detection of severe morbidity. It built on previous research that showed gender differences in food preparation/food and drinking habits and thus exposure to liver fluke infection.

Although with some limitations when it came to addressing the issue of sex and gender, the research protocol included a commitment to the collection of sex-disaggregated data, but there was no further specification on exploring the wider potential effects of sex/gender in the project, nor to the need for sex- and gender-specific strategies to ensure sufficient numbers of females were included in the sampling. Nonetheless, the sex-disaggregated data already provided some important insights in this socio-cultural context; for example, they found that women showed a greater uptake of liver fluke control activities than men, both in the initial inclusion in the project and in follow-up examination once a pathology had been diagnosed. On its own, these findings are not sufficient to make any wider interpretations other than the need for a gender analysis.

The two following case studies went one step further. The collection and analysis of sex- and gender-disaggregated data–as shown in the case study above–is always the very first step and public health scientists have historically been reasonably fastidious at initial disaggregation, but all too quick to re-aggregate as the soonest possible moment, typically to draw higher impact of the burden and effects of any disease or health event. Sex, or sex as a biological variable (SABV), have become a policy-level requirement at the National Institute of Health of the United States of America. However, robust intersectional gender analysis processes demand more than simple binary disaggregation and are typically neglected as part of good practice in public health research [22]. Sex and gender analysis is part of the entire research process and should be an ongoing dimension of multivariate analysis and overarching conceptual thinking around research questions. Hence, building gender into the design and methodology of a research protocol is one-step along the path of integrating gender in data capture, but implementation of an analysis plan and interpretation of the implications is part of the entire research process. Furthermore, gender intersects with many other axes of inequality and privilege that are critical to a holistic understanding of the determinants of health and disease [22]. As such, gender cannot be considered in isolation. We examine how public health researchers make sense of these intersections to produce more robust knowledge to address core questions, even though the projects are not designed as gender projects *per se*. The next two examples are from projects that included gendered dimensions of public health research from either the design stage or at least when considering the methodology.

### Health systems governance for equitable social health protection in Tanzania

In the context of the global drive to achieve social health protection through universal health coverage, this r4d public health project focused on health systems sought to increase understanding of how to identify excluded populations and facilitate their access to health services, and how to leverage health systems governance to make social health protection schemes more inclusive. The initial design of the study did not fully integrate the gender equality dimension, meaning that the research objectives were not sensitive to capture how the health needs of men and women are integrated in the existing health financing strategy, nor how the social health protection schemes in Tanzania have been designed to address those needs. To mainstream the gender equity in the r4d study of health system’s governance on social health protection, it was retrospectively decided to include gender-related questions in the interview guide, e.g. “are men and women able to access the health insurance scheme equally?”. Additionally, looking into the extent to which women were working in informal positions that did not afford any social health protection, rather than formal employment, the experience of women-led households in terms of accessing benefits in case of a claim, etc. The findings reflected how social protection systems often did not help them cope with risks, recover from shocks potentially compounding situations of poverty or vulnerability.

### Health impacts of large natural resource extraction projects in Ghana and Tanzania on local communities

This multi-site r4d project took place in Burkina Faso, Ghana, Mozambique and Tanzania and aimed at promoting health impact assessment in Africa, particularly in relation to natural resource extraction projects [23, 24]. In this manuscript, we focus on the experience of the Tanzanian and Ghanaian study sites. Within the overall frame of the project, a qualitative study was conducted to explore the perception of local communities on health impacts of large natural resource extraction projects [24, 25].

The qualitative study included a specific analysis focusing on gendered health impacts as perceived by communities living around three industrial gold mines in Tanzania [26]. Because there was a clear commitment to identify gender differences to ensure that the recommended policies and practice from the “health impact assessment for promoting sustainable development” (HIA4SD) project would be robust and to enable participants to express their opinions openly sessions were held with women and men separately. Typically, single-sex/gender groups are used to overcome the gendered dynamics of mixed-sex/gender focus group discussions. By using a participatory approach, different groups of women and men were asked the same questions about the positive and negative impacts of mining on the wider determinants of health. This revealed that there are “gendered” impacts that affect men and women differently due to their distinct social roles and occupations in the community. Focus discussions were explicitly scheduled at times that were most convenient to each gender role to increase chances on participation. It was important to be flexible in the research process and adapt to the gender-related issues that arise, particularly during the data collection phase. To remain gender sensitive during the project implementation, the field activities were run by a gender-balanced team. Having a research team composed of both genders increased the chances that the team was (i) aware of the possible gender differences in response, (ii) culturally sensitive to the gender related issues and (iii) was committed to identifying these gender differences.

In addition to gender-separated focus group discussions with community members, in Ghana, we conducted key informant interviews with community leaders, health sector leaders and leaders of natural resource extraction projects. Similar to global trends, which show a disproportionately lower number of women in leadership, less than 20% of the leaders were women. In order to compensate for this, the team had to adapt the research design to increase women in the pool of respondents. One observation worth mentioning is that when queen mothers (members of the royal family of each town and village) were invited to the interviews with the traditional leaders, they tended to talk far less than when interviewed alone. Possibly, this is attributable to the differences in power-relationships in the communities. An understanding of this is essential in designing interventions and solutions for improvement. We noticed that addressing gender issues that arise in the research process, requires sensitivity and awareness of the socio-cultural underpinnings of some of the observations and an understanding of how to navigate them.

### Resistances and opportunities

This theme is transversal to the two previous themes and it relates to the attitudinal challenge, poor knowledge and negative attitudes towards gender in public health research that limit gender integration in design, methodology and analysis. Convincing principal investigators and public health scientists of the potential value and health impact of gender analyses remains an attitudinal challenge in the public health sciences where gender is less well understood as a significant dimension of health status, even in quantitative, clinical and epidemiological studies. In the following example, the emergence of glaring gender issues around representation and sampling became the driving issue around which attitudes to gender within the project mobilized and retrospective corrective actions introduced.

### Surveillance and response to zoonotic diseases in Maya communities of Guatemala: A case for one health

This first research project is an example of having a reverse or backwards adaptation of the project design to become more gender-responsive. The main goal of this project was to understand the burden of brucellosis and leptospirosis in a remote area of Guatemala characterized by extreme poverty, poor access to health services and a plurimedical system. The project was designed as an intercultural transdisciplinary process that brought together government officials from the animal and human health ministries, the private sector, local communities, local indigenous authorities (Councils of Elders) and researchers, to jointly develop robust solutions for local health programs and influence national policy towards incorporating a One Health approach. The main issue addressed in the project design was how to build a resilient, truly participatory partnership amidst historical mistrust and great sociolinguistic and cultural diversity, which meant a co-existence of multiple knowledge systems with vastly different levels of power and agency. The level of effort needed to achieve an intercultural approach relegated gender considerations to the backstage, where the interdisciplinary academic team did not propose a specific approach to address gender issues at the start-up phase. This error became painfully obvious after the first project evaluation, one year into the project, showing an enormous gender gap in the under representation of women from the local communities and zero representation of indigenous women from the Councils of Elders. In all community planning meetings, for example, mostly just men showed up, making decisions that later evidenced the lack of knowledge they had on many of the household practices directly affecting the lives of children, women and their animals. Anthropologists conducted deep ethnography while living with local families, confirming that almost all decisions directly linked to the use of the surveillance system, the biomedical sample collection of sick patients, the adherence to treatment and, most importantly, the implementation of preventive measures, were made by women. Unless they were involved in the planning, validation and implementation of all project activities, it became evident the project was not going to be successful.

The social science team proposed a ‘backward planning’ exercise that started from understanding what outcomes were desired by all stakeholders. Given no indigenous women were chosen by community leaders to integrate the transdisciplinary working group, their voices were absent and had to be included by holding additional interviews and workshops in their communities. From the ‘enhanced’ goals that now held a balanced gender view, the team analysed the underlying assumptions to reach them, making visible initially overlooked contextual problems that needed to be addressed and changed through the research intervention. This project set to change these excluding dynamics by implementing new activities and conditions that could modulate negative factors. The transdisciplinary approach was equipped with tools to promote an adaptive process, since it defined from the onset that initial plans (co-defined with all stakeholders), were to be formally revised in annual sessions to redirect resources, methods and activities as needed. As such, the project introduced the concept of “gender equity” through the second year workshops and requested community representatives to include at least 25% of women in all meetings, as well as to appoint at least one female leader per town. Support and training was given to the new female leaders throughout the remaining project life to build self-confidence and break initial resistance. Reflexive exercises were held to show how men and women understood household dynamics differently and how complementing views produced more robust designs in the syndromic surveillance platform and in the communication and education campaigns to be designed. These interventions increased and social robustness of the interventions. One particularly relevant example was the adaptation of the original design of the household syndromic surveillance system to respond to women’s requests for change, which increased its use threefold, yielding more data for the epidemiology study and augmenting community buy-in.

In support of overcoming overlapping layers of exclusion, the project addressed language, distance and poverty barriers that affected women more directly. It started including equipment (individual earphones) to have permanent onsite translation done by professional linguists, allowing immediate communication between English, Spanish and Q’eqchi’ speakers. Glossaries of complex terms were prepared ahead of time to facilitate the explanation in Q’eqchi’ of novel terminology such as ‘epidemiology’, ‘gender’, or ‘lab tests’, as well as to explain complex Maya terms related to traditional worldviews important to the discussions. In this way, ethnolinguistic groups in general and women in particular felt represented more equally and were more willing to voice concerns, propose new activities and engage in implementation procedures. Women were also paid a stipend to cover their travel and opportunity costs for attending meetings, same as men, and were allowed to bring their small children to the workshops. They were offered overnight room and board for them and their accompanying children so they could remain participating in the entire workshop and not have to leave half way due to public transportation limitations. Non-Maya attendees received cultural awareness training to accept open breast-feeding during meetings, the presence of children’s games and baby hammocks, as well as to allocate specific time slots to encourage women to give their opinions. Overall, the project was able to achieve women’s buy-in and increased their participation to have a real impact on the projects’ outcomes. As an example, in the third year of the project, the local community committees created to design and implement education and communication campaigns to prevent zoonotic disease transmission were two thirds female. This composition prompted creative interventions in the form of community fairs, drama and poetry contests for kids, cooking workshops for producing brucella-free dairy products, and other activities clearly belonging to the domain of women.

## Discussion

This collective retrospective meta-synthesis highlighted the importance of including the issue of gender in the conceptualization and design of all public health projects; all researchers from each of the case studies included in this article agreed that not doing so was an important drawback. It was a consensus that, even when, apparently, projects do not hypothesize gender dimensions to their analysis and findings, the capture of gender data is fundamental in public health research.

Historically, and a finding echoed in the research projects reviewed in this paper, the gender and sex dimensions of global and public health research have been eschewed by what have been viewed as more pressing and impactful variables in data collection and analysis such as socio-economic status captured with quantitative method and survey tools. An equitable (gender, ethnic or other) process in a research for development project does not happen naturally, it is a carefully planned and designed intervention that needs monitoring and evaluation to implement timely corrective measures and address conflict constructively. However, so often, the reaction to the question “and gender?” receives a defensive response that there are no specific gender imbalances in relation to a given disease, unless that is, the health issues is sexual and reproductive and/or women’s health. Such attitudes were also experienced across all the projects engaged in this reflective post-research process and members of our respective teams employed a number of strategies to gain traction to include gender dimensions to the research projects. Convincing principle investigators and public health scientists of the added value and health impact of including gender in the design across qualitative and quantitative, clinical and epidemiological studies, and conducting gender analyses remains an attitudinal challenge in some areas of the public health sciences where gender is less well understood as a significant dimension of health status.

It is now known, and our case studies confirmed, that the issue of gender should be considered in all phases of a research project. Researchers make sure they take into account who participates as respondents, when data is collected and where, who is present, who collects data, who analysis data [1]. Sex- and gender-disaggregation can serve as starting point to showing female-male differences that can trigger further investigation of how gender power relations are constituted and negotiated within the communities and the health systems, how they can create inequities; but also reflecting on how the research itself is embedded within potential power relations (who collects the data, who responds, etc.) [1, 27]. According to Morgan and colleagues, to understand gender power relations, it is fundamental to explore “who has what” (access to resources), “who does what” (the division of labour and everyday practices), “how values are defined” (social norms) and “who decides” (rules and decision-making).

Additionally, gender frameworks, which provide a structure for organizing information about gender roles and relations, can allow for a more organized process, helping researchers through identifying research questions, and planning data collection and data analysis. Having gender analysis questions can then allow researchers to go beyond identifying the differences between men and women and respond to “why?” the identified power relations cause inequities. One useful tool to prevent our research from missing out important gender-related information, particularly for those who lack a background in gender studies, is the “Gender checklist” created by Hardy and colleagues [28]. This checklist aims at supporting researchers in ensuring their studies are gender sensitive, across all project time points.

Moving beyond collecting sex- and gender-disaggregated data, in the research design and research process researchers should also consider gender power and participation. At this stage, the first step is making sure there is an equal balance of men and women as study participants. Second, it is critical to take into account when and where data is collected as, for example, women may not feel comfortable speaking openly about certain topics in a place where male participants are also present. In this sense, it is also important to make sure that whoever is present, even if part of the research team, does not compromise the information that is shared by participants. Finally, the data analysis could also be influenced by the researchers own gender biases. Hence, it is necessary to consider this during the analysis of data. In conclusion, having a diverse research team, increases the chances of identifying power differentials quickly and contributing towards gender equity.

In circumstances where researchers did not take the issue of gender into account early on, the process of backward planning, as seen in the One Health project in Guatemala for instance, can help research teams identify initially overlooked gaps that could harm project outcomes, and lead them in designing targeted activities to promote needed change. An initial gender imbalance can be corrected through adaptive measures in the course of a project, which can impact positively project implementation, increase buy-in by making interventions gender responsive, and overall produce more intersectionally-robust outcomes. Agile transdisciplinary project designs can rectify omissions or gender-blind projects during the implementation process, even once data collection has commenced, by taking a step back to reflect on the gender dimensions of the project as a whole and by actively making changes to respond.

Our case studies revealed that gender interplays with other axes of inequality, such as age, disability and geographical location; hence, intersectionality is important for inclusive and responsive programmes/global health interventions towards health equity. Cross-sectoral collaborations across social, environmental and health sciences are critical to effectively use the data in view of an evidence- and development-based research agenda. Previous studies have recommended having representation from domain experts and gender scholars, survey designers and analysts, and community partners and policy makers in order to establish data systems that enable studying health at the intersection of gender and other social determinants (e.g. race, religion, and social class) [29].

Through our peer-interviews and research case studies, we identified several lessons learnt to guide and improve the design and process of conductive gender attentive research in global and public health. These are of particular importance to produce health and social outcomes that contribute to improving the health and status of women by addressing and contributing to gender equity [30]. By conducting health research with a gender lens and bringing forth the various background dimensions that interact to create layers of inequality in which the role and position of women and men are embedded, a more complete analysis can be developed to better capture the ways in which public policy is experienced by various groups [31]. Furthermore, conducting gender attentive or responsive research provides the opportunity for policy makers to improve their understanding of gender and its interfaces with other social determinants (class, race, nationality, income, etc.) to develop the right approaches and make more responsive and efficient recommendations to address gender equality in public policy developments and decision making.

## Conclusions

In this paper, we have outlined and discussed the experiences of addressing gender in four global public health research projects. We have made the argument that it is critical to have a gender lens to all data being collected from the very early stage of conceptualization of a research study in order to be actively incorporated into the design of a research programme and to conduct in-depth gendered analysis, in order to better capture the multiple layers where gender issues should be considered (team composition, research design, data collection and analysis). We have also shown that it is possible to rectify and course-correct research projects that did not design for gender in their original proposals, and that a retrospective reflexive step even during a project can go some way towards producing gender-responsive findings.

Gender attentive health research allows to generate more evidence to inform policy frameworks and related guidelines to make sure our projects are not gender blind (and do no harm), and to address gender inequities, discrimination or exclusion through subsequent health programmes, strategies or interventions in a transformative way.

Gender in public and global health matters and needs to be more effectively addressed by the research and policy community, all the while considering its intersectionality to produce more robust knowledge on the core questions to achieve gender and health equity. Addressing gender in public and global health projects as integral part of the research design and the inclusion of gendered analysis are inevitable in the strive towards more equitable health systems towards reaching the SDGs, in particular SDG 3 good health and wellbeing and SDG 5 on gender equality.

## Supporting information

S1 Table Interview guidelines of the synthesis initiative. (DOCX)

S2 Table Webinar questions. (DOCX)
