# Supplementary material for: Gender in public health research: Reflections on design and process across four research projects in low-and middle-income countries
Source: PLOS Glob Public Health. 2023 Apr 12;3(4):e0000808. doi: 10.1371/journal.pgph.0000808 (PMC10096266; doi:10.1371/journal.pgph.0000808)
Supplement: S1 Table — (DOCX) [file pgph.0000808.s001.docx]

**S1 Table.** Interview guidelines of the synthesis initiative.

| Results | 1. What are the **key research results** from your projects that are of relevance to **UHC policy and practice**? |
| --- | --- |
| Involvement of policy makers | 2. Were national or international **policy makers** involved in your **project/research design**?   - - If so, who, how and at what stage?   - Have you organized any stakeholder workshop? If so, who has participated?   - If not, have you already identified national or international policy makers that could be interested by your research results? How?   3. Does your research evidence (already) **play a role in national (or international) policymaking**,   - - If so how? Best practice examples? |
| Dissemination | 4. Do you have a **dissemination plan** as part of your project?   - - If so, what have you planned? Are you on track, facing challenges? Has your dissemination plan as part of the initial proposal been playing a role?   - Do you have any resources allocated to dissemination?   - Are policy makers a target group of your dissemination activities?   - Are there **any obstacles** to disseminate evidence from the research area to the policy/decision sphere? |
| Gender | 5. How were **gender** aspects addressed by your research project?   - - Did you differentiate between gender neutral, -sensitive, -specific and transformative approaches?   - Did you collect sex-disaggregated data?   - Are there any best practice examples you could share?   - How can they be effectively translated to policy and practice?   - Are there any obstacles to share? |
| Social inclusion | 6. How were **social inclusion/vulnerability** aspects addressed by your research project?   - - Are there any best practice examples you could share?   - How can they be effectively translated to policy and practice? |
