# Supplementary material for: Gender in public health research: Reflections on design and process across four research projects in low-and middle-income countries
Source: PLOS Glob Public Health. 2023 Apr 12;3(4):e0000808. doi: 10.1371/journal.pgph.0000808 (PMC10096266; doi:10.1371/journal.pgph.0000808)
Supplement: S2 Table — (DOCX) [file pgph.0000808.s002.docx]

**S2 Table.** Webinar questions.

| Research design | 1. Did you think about gender when your project was being conceived and then implemented?  - What steps did you take to give gender issues adequate attention, if at all? - And/or, have there been any issues that have been considered especially related to women or to men specifically? |
| --- | --- |
| Research process | 1. Are all your data sex-disaggregated? If so, are there opportunities for analysis of these? Have data that is sex-disaggregated shown any patterns? |
| Resistances and opportunities | 1. Have gender been issues that have arisen in your project? And/or are inequalities and inclusion relevant to your research? 2. What, if any, resistance is there to taking up questions of sex and gender – and if so how did you overcome them? What are the challenges? |
